# Control of diabetes, hypertension, and dyslipidemia in Jordan: a cross-sectional study

**DOI:** 10.1097/MS9.0000000000000272

**Published:** 2023-03-24

**Authors:** Dana Hyassat, Nancy Abu Noor, Qais AlAjlouni, Yazan Jarrar, Raed Qarajeh, Awn Mahasneh, Zaid Elzoubi, Yousef Khader, Oraib Farahid, Mohammed El-Khateeb, Kamel Ajlouni

**Affiliations:** a The National Center for Diabetes, Endocrinology and Genetics, University of Jordan Amman, Amman; b Jordan University of Science and Technology (JUST), Al Ramtha, Irdid, Jordan

**Keywords:** associated factors, blood pressure, dyslipidemia, prevalence, type 2 diabetes mellitus

## Abstract

To determine the level of glycemic, blood pressure (BP), and lipids control among patients with type 2 diabetes mellitus (DM) attending the National Center for Diabetes, Endocrinology and Genetics and to determine factors associated with poor control.

**Methods:**

A cross-sectional study of 1200 Jordanian type 2 DM patients was included in this study during the period of December 2017–December 2018. We reviewed the charts of these patients until January 2020. Data obtained from medical records included information about sociodemographic variables, anthropometric measurements, glycated hemoglobin (HbA1c), BP, low-density lipoprotein (LDL), the presence of DM complications, and treatment.

**Results:**

The percentage of subjects who had HbA1c values of less than 7% was 41.7%. BP targets (<140/90 and 130/80 mmHg) were achieved in 61.9 and 22% of our patients, respectively. LDL targets less than 100 and 70 mg/dl or less were achieved in 52.2 and 15.9% of our studied population. Only 15.4% of our patients could have simultaneous control of HbA1c less than 7%, BP less than 140/90 mmHg, and LDL less than 100 mg/dl. Factors associated with poor glycemic control were obesity [odds ratio (OR)=1.9], DM duration between 5 and 10 years or more than 10 years (OR=1.8 and 2.5, respectively), and the use of a combination of oral hypoglycemic agent plus insulin or insulin alone (OR=2.4 and 6.2, respectively). Moreover, factors associated with uncontrolled BP (≥140/90) were male gender (OR=1.4), age 50–59 years or at least 60 years (OR=3.3 and 6.6, respectively), overweight and obesity (OR=1.6 and 1.4, respectively), insulin use (OR=1.6), and LDL at least 100 mg/dl (OR=1.4).

**Conclusion:**

The overall prevalence of poor glycemic control was high and alarming. Future research should focus on capturing all variables that may impact glycemic, BP, and dyslipidemia control, with special emphasis on a healthy lifestyle that would be of great benefit in this control.

Highlights

- Good glycemic control (HbA1c<7%) was achieved in 41.7% of our patients.
- Blood pressure targets (<140/90 and 130/80) were achieved in 61.9 and 22% of our patients, respectively.
- Low-density lipoprotein targets less than 100 and 70 mg/dl or less were achieved in 52.2 and 15.9% of our studied population, respectively.
- The simultaneous control of glycated hemoglobin (<7%), blood pressure (<140/90), and low-density lipoprotein (<100 mg/dl) collectively known as the ABCs of diabetes was achieved in 15.4% of our patients.

## Introduction

Diabetes mellitus (DM) is a major, rapidly growing health concern that has reached alarming figures. In 2019, nearly half a billion people worldwide were living with diabetes. The estimated number of people (20–79 years) living with diabetes has increased by 62% during the past 10 years, from 285 million in 2009 to 463 million nowadays^1^. The highest world-age-standardized diabetes prevalence is in the Middle East and North Africa region, where 12.2% of their population is estimated to have diabetes^1^. In Jordan, the overall age-standardized prevalence of diabetes increased from 13% in 1994 to 17.1% in 2004, 22.2% in 2009, and 23.7% in 2017, foreboding future growth in premature morbidity as well as mortality and mounting huge socioeconomic and healthcare costs^2^.

DM is a leading cause of various complications such as blindness, cerebrovascular disease, and end-stage kidney disease, which are 2–5 times more common in patients with diabetes^3^. The United Kingdom Prospective Diabetes Study and the Diabetes Control and Complications Trial found that the risk of diabetes complications can be reduced by intensive glycemic, blood pressure (BP), and lipid control. Therefore, successful management of risk factors such as diabetes, hypertension, and dyslipidemia is the benchmark of good clinical care^4^,^5^. The American Diabetes Association (ADA) recommends that most adults with diabetes achieve glycated hemoglobin (HbA1c) less than 7%, low-density lipoprotein cholesterol (LDL-C) less than 100 mg/dl, and BP less than 140/90 mmHg^6^, while the American Association of Clinical Endocrinologists (AACE) had further recommended HbA1c of less than 6.5% as a target glycemic goal^7^.

There is strong evidence of the beneficial effect of simultaneous control of (the ‘ABCs’) HbA1c, BP, and LDL-C in reducing diabetes complications and death; multiple studies have shown that achievement of all three goals together were low (10–30%)^8^–^12^. In a previous study in Jordan conducted in 2009, 56, 47, and 10.4% of type 2 DM patients could achieve HbA1c less than 7%, BP less than 130/80, and LDL-C less than 2.6 mmol/l^13^.

In Jordan, there are limited data on achieving targets for diabetic patients with respect to HbA1c, BP, and lipid profile. This study was conducted to determine the level of glycemic, BP, and lipids control among patients attending the National Center for Diabetes, Endocrinology, and Genetics (NCDEG) and to determine factors associated with poor control.

## Methods

### Study design and study population

A cross-sectional study of 1200 patients with type 2 DM was conducted at the NCDEG in Amman–Jordan, during the period from December 2017 to December 2018. We reviewed the charts of these patients until January 2020. All patients with type 2 diabetes (aged>25 years) and had at least three readings of HbA1c, BP, and lipid profile documented in the medical records were included in the study. Patients with type 1 DM and women with gestational DM were excluded. We have used each of the three consecutive measured HbA1c to determine the glycemic control of the patients. Data were collected by trained nurses and general practitioners. Patients’ sociodemographic data including gender, age, marital status as well as educational level; anthropometric and clinical characteristics including weight, height, BMI, waist circumference, presence of other comorbidities such as hypertension, dyslipidemia, and hyperuricemia; as well as diabetes complications (retinopathy, nephropathy, and neuropathy). Additional data, including smoking history, type, and duration of diabetes treatment, were all abstracted from medical records. The data collectors were trained in the abstraction of data from medical records and ensuring data confidentiality.

### Variables definition

In the current practice of the NCDEG, anthropometric measurements, including weight, height, and waist circumference, were measured while the subjects were wearing light clothing and no shoes. Waist circumference was estimated at the end of a normal expiration using a nonstretchable tape held in a horizontal plane around the abdomen at the level of the iliac crest. It was considered normal if the waist was between 88.5 and 91.8 cm in men and from 84.5 to 88.5 cm in women according to anthropometric cutoff values for detecting metabolic abnormalities in Jordanian adults^14^. Waist-to-height ratio was considered elevated if it was greater than 0.5. BMI was calculated by dividing weight in kilograms by height in meters squared. Patients were classified according to BMI following the recommendation of the WHO as adopted by the ADA^15^. Readings of systolic and diastolic BPs were taken while the subjects were seated, and the arm was kept at the heart level after at least 5 min of rest, using a standardized mercury sphygmomanometer, high BP was defined as systolic BP at least 130 mmHg or diastolic BP at least 80 mmHg or if the patient was already on antihypertensive drugs^6^. Controlled BP was divided into two categories: either BP less than 130/80 mmHg or BP less than 140/90 mmHg.

Metabolic abnormalities were defined according to the American Diabetes Association 2017^6^ as follows: total serum cholesterol at least 200 mg/dl, serum LDL at least 100 mg/dl, serum triglyceride at least 150 mg/dl, serum high-density lipoprotein (HDL) 40 mg/dl or less in men, and 50 mg/dl or less in women, or if the patient was already on antidyslipidemic agents. Smoking was classified into three categories according to WHO guidelines 1998^16^.

DM was diagnosed if the patient had a fasting plasma glucose 126 mg/dl or less (7.0 mmol/l) on two occasions or if the patient had random plasma glucose 200 mg/dl or less (11.1 mmol/l) in the presence of classic symptoms of hyperglycemia, or if he or she had HbA1c 6.5% or less. Moreover, diabetes was considered to be controlled if the patient had HbA1c less than 7.0% according to the ADA (2017) guidelines^6^, or HbA1c less than 6.5% according to the American Association of Clinical Endocrinologists (AACE 2015)^7^.

Retinopathy was diagnosed if it was documented by either the ophthalmologist or the treating physician in the medical records or if the patient had received laser treatment. Neuropathy was diagnosed if there were any of the following symptoms (numbness, tingling, or pain in toes, feet, legs, hands, arms, and fingers) in the patient’s medical records or if the patient had done a Nerve Conduction Study which proves the presence of diabetic neuropathy or if the patient was receiving treatment for the above condition.

The present study was approved by the National Centre for Diabetes, Endocrinology and Genetics (NCDG) Ethics’ Committee. Identifying information was kept strictly confidential and the data were used only for scientific purposes by the researchers.

### Statistical analysis

Data were entered and analyzed using the Statistical Package for Social Science (SPSS version 20). Data were examined initially for data entry errors and outlying values. Any detected errors were corrected as appropriate. In univariate analysis, continuous variables were analyzed as mean±SD and categorical variables as frequencies and percentages. Percentages were compared using *χ* ^2^ test. Multivariate analysis using binary logistic regression analysis was conducted to determine factors associated with poor glycemic control, high LDL-C, and uncontrolled BP. A *P* value of less than 0.05 was considered statistically significant.

## Results

### Participants’ characteristics

This study included 1200 type 2 diabetic patients, aged between 19 and 87 years, with a mean age (SD) of 55.2 (9.9) years. Fifty-one percent of patients were males, 58% of them were obese, and 33% were overweight. Seventy-five percent of patients had hypertension, while 81% of them had dyslipidemia. Nine percent of diabetic patients had neuropathy and 20% had retinopathy, 39% of the patients were on the combined oral hypoglycemic agent (OHA) and basal insulin. The sociodemographic and clinical characteristics of the study population are presented in Table 1.

**Table 1:** The sociodemographic and clinical characteristics of T2DM patients according to gender

Variables	Total, *N* (%)
Age (years)	
<40	90 (7.5)
40–49	232 (19.3)
50–59	480 (40.0)
≥60	398 (33.2)
Gender	
Females	586 (48.8)
Males	614 (51.2)
Educational level	
Less than high school	638 (53.6)
More than high school (diploma+university)	552 (46.4)
Smoking	
Smoker	291 (24.3)
Nonsmoker	909 (75.7)
Marital status	
Married	1077 (89.7)
Never-married	123 (10.3)
Duration of diabetes (years)	
<5	438 (36.5)
5–10	359 (29.9)
>10	403 (33.6)
BMI (kg/m^2^)	
Normal	111 (9.3)
Overweight	393 (32.7)
Obese	696 (58.0)
Increased waist circumference (cm)^a^	1066 (88.8)
Increased waist-to-height ratio^b^	1152 (96.0)
Hypertension^c^	905 (75.4)
Dyslipidemia^d^	969 (80.8)
Neuropathy	109 (9.1)
Retinopathy	239 (19.9)
Renal impairment	204 (17.0)
Hyperuricemia	208(17.3)
Treatment	
OHA	697 (58.1)
OHA+insulin	472 (39.3)
Insulin	31 (2.6)

Categorical variables are expressed as frequencies and percentages.

^a^ Increased waist circumference if the waist was between 88.5 and 91.8 cm in men and from 84.5 to 88.5 cm in women.

^b^ Waist-to-height ratio was considered elevated if it was >0.5.

^c^ High blood pressure (BP) if systolic BP ≥130 mmHg or diastolic BP ≥80 mmHg or if the patient was already on antihypertensive drugs.

^d^ Dyslipidemia if total serum cholesterol ≥200 mg/dl, serum LDL ≥100 mg/dl, serum triglyceride ≥150 mg/dl, serum HDL ≤40 mg/dl in men, and ≤50 mg/dl in women, or if the patient was already on antidyslipidemic agents.

HDL, high-density lipoprotein; LDL, low-density lipoprotein; OHA, oral hypoglycemic agent; T2DM, type 2 diabetes mellitus.

### Glycemic control

Table 2 shows the percentage of subjects who had HbA1c values of less than 6.5%, less than 7%, 7.5% or less, less than 8% and at least 9%. The percentages were 24.4% for HbA1c less than 6.5%, 41.7% for HbA1c less than 7%, 58.8% for HbA1c 7.5% or less, 72.4% for HbA1c less than 8% and 89.4% for HbA1c 9% or less.

**Table 2:** The glycemic, blood pressure, and blood lipids control among T2DM patients according to gender

Variables	Female, *N* (%)	Male, *N* (%)	*P* *	Total, *N* (%)
HbA1C (%)				
** **<6.5	125 (21.5)	166 (27.2)	0.022	291 (24.4)
** **<7.0	223 (38.3)	275 (45.0)	0.056	498 (41.7)
** **≤7.5	322 (55.3)	379 (62.0)	0.028	701 (58.8)
** **<8.0	415 (71.3)	449 (73.5)	0.400	864 (72.4)
** **≤9	529 (90.7)	544 (88.9)	0.291	1073 (89.4)
Blood pressure (mmHg)			0.325	
** **Controlled (<140/90)	370 (63.4)	369 (60.6)		739 (61.9)
** **Uncontrolled (≥140/90)	214 (36.6)	240 (39.4)		454 (38.1)
Blood pressure (mmHg)			0.006	
** **Controlled (<130/80)	149 (25.5)	115 (18.9)		264 (22.1)
** **Uncontrolled (≥130/80)	435(74.5)	494 (81.1)		929 (77.9)
Total cholesterol (mg/dl)			0.191	
** **<200	237 (83.2)	255 (87.0)		492 (85.1)
** **≥200	48 (16.8)	38 (13.0)		86 (14.9)
LDL-C (mg/dl)			0.086	
** **<100	196 (49.1)	224 (55.2)		420 (52.2)
** **≥100	203 (50.9)	182 (44.8)		385 (47.8)
LDL-C (mg/dl)			0.005	
** **≤70	49 (12.3)	79 (19.5)		128 (15.9)
** **>70	350 (87.7)	327 (80.5)		677 (84.1)
LDL-C (mg/dl)			0.261	
** **<130	321 (80.5)	339 (83.5)		660 (82.0)
** **≥130	78 (19.5)	67 (16.5)		145 (18.0)
HDL-C (mg/dl)			0.019	
** **F>50, M>40	102 (30.7)	130 (39.4)		232 (35.0)
** **F≤50, M≤40	230 (69.3)	200 (60.6)		430 (65.0)
Triglycerides (mg/dl)			0.699	
** **<150	217 (53.2)	212 (51.8)		429 (52.5)
** **≥150	191 (46.9)	197 (48.2)		388 (47.5)

* Comparisons were made using *χ* ^2^ tests. A *P* value of <0.005 was considered statistically significant.

HbA1C, glycated hemoglobin; HDL-C, high-density lipoprotein cholesterol; LDL-C, low-density lipoprotein cholesterol; T2DM, type 2 diabetes mellitus.

### BP control

The percentage of subjects who had controlled BP, defined as BP less than 140/90, was 61.9%, with no statistically significant difference between males and females.

### Dyslipidemia control

Concerning lipid profile, 85.1%, 52.5%, and 35% of patients, respectively, had total cholesterol less than 200 mg/dl, triglycerides level less than 150 mg/dl, and HDL-cholesterol (C) level higher than 50 mg/dl in females or higher than 40 mg/dl in males. When LDL-C level less than 100 mg/dl was taken as a target level, the percentage of patients who achieved this level was 52.2%, but upon considering LDL-C level 70 mg/dl or less as the optimal LDL-C level needed to be reached, only 15.9% of patients achieved this recommended target.

### Simultaneous control of HbA1c, BP, and LDL

The percentage of patients who met the three ADA targets (HbA1c level <7%, BP <140/90, and LDL-C<100 mg/dl) was 15.4%, with no difference between males and females (*P*=0.916).

### Factors associated with poor glycemic control

Using multiple logistic analysis, patients between 50 and 59 years old or at least 60 years old were less likely to have poor glycemic control than those who were less than 50 years old. Males were less likely to have poor glycemic control than females (*P*=0.027). Obese patients were 1.9 times more likely to have uncontrolled DM (*P*=0.006), patients with DM duration between 5 and 10 years or more than 10 years were 1.8 times and 2.5 times more likely to have poor glycemic control compared to those with DM duration less than 5 years (*P*=0.001 and 0.002, respectively). Patients who were on either combination of OHAs and insulin or insulin alone were 2.4 and 6.2 times more likely to have poor glycemic control than patients who were taking only OHAs (*P*=0.039 and 0.001, respectively) as shown in Table 3.

**Table 3:** Factors related to poor glycemic control (HbA1c ≥7.0%) among T2DM patients using multivariable logistic regression

Variable	OR	95% CI	*P*
Gender			
** **Female*	1		
** **Male	0.7	0.6–0.9	0.027
BMI			
** **Normal*	1		
** **Overweight	1.2	0.7–1.9	0.497
** **Obese	1.9	1.2–3.1	0.006
Duration of diabetes (years)			
** **<5*	1		
** **5–10	1.8	1.3–2.5	0.001
** **>10	2.5	1.7–3.6	0.002
Age (years)			
** **<40*	1		
** **40–49	0.9	0.5–1.6	0.763
** **50–59	0.5	0.3–0.9	0.020
** **≥60	0.4	0.2–0.8	0.004
Treatment			
** **OHA	1		
** **OHA+insulin	2.4	1.1–5.5	0.039
** **Insulin	6.2	4.5–8.4	0.001

* Reference group, a *P* value of <0.005 was considered statistically significant.

HbA1C, glycated hemoglobin; OR, odds ratio; OHA, oral hypoglycemic agent; T2DM, type 2 diabetes mellitus.

### Factors associated with LDL level

Table 4 shows the multivariate analysis of factors associated with high LDL. Males were more likely to have LDL-C level at least 100 mg/dl (OR=1.3; 95% CI: 1.1–1.5), whereas people with age at least 50 years or older were less likely to have LDL-C level at least 100 mg/dl.

**Table 4:** Factors associated with LDL among T2DM patients using multivariable logistic regression

Variable	OR	95% CI	*P*
Gender			
** **Female*	1	1.1–1.5	0.035
** **Male	1.3		
Age (years)			
** **<40*	1	0.5–1.6	0.277
** **40–49	0.7	0.3–0.9	0.024
** **50–59	0.5	0.2–0.8	0.004
** ≥**60	0.4		

* Reference group, a *P* value of <0.005 was considered statistically significant.

LDL, low-density lipoprotein; OR, odds ratio; T2DM, type 2 diabetes mellitus.

### Factors associated with uncontrolled BP

As shown in Table 5, males were 1.4 times more likely to have uncontrolled BP than females (*P*=0.034). Ages between 50 and 59 years or at least 60 years were 3.3 and 6.6 times, respectively, to have uncontrolled BP than those less than 50 years old (*P*=0.001 and 0.001, respectively). Patients who were overweight or obese were 1.6 and 1.4 times more likely to have uncontrolled BP (*P*=0.005 and 0.045, respectively). Insulin users were 1.6 times more likely to have uncontrolled BP than other treatment groups (*P*=0.001). Patients with LDL-C level higher than 100 mg/dl were 1.4 times more likely to have uncontrolled BP compared to those with LDL-C less than 100 mg/dl (*P*=0.045) (Table 5).

**Table 5:** Factors related to blood pressure among T2DM patients using multivariable logistic regression

Variable	OR	95% CI	*P*
Gender			
** **Female*	1		
** **Male	1.4	1.02–1.91	0.034
Age (years)			
** **<40*	1		
** **40–49	1.7	0.8–3.7	0.148
** **50–59	3.3	1.7–6.7	0.001
** **≥60	6.6	3.3–13.4	0.001
BMI			
** **Normal*	1		
** **Overweight	1.6	1.1–3.8	0.005
** **Obese	1.4	1.2–4.1	0.045
Treatment			
** **OHA	1		
** **OHA+insulin	0.7	0.3–0.9	0.039
** **Insulin	1.6	1.1–2.1	0.001
LDL-C			
** **<100*	1		
** **≥100	1.4	1.0–3.8	0.045

* Reference group, a *P* value of <0.005 was considered statistically significant.

LDL-C, low-density lipoprotein cholesterol; OHA, oral hypoglycemic agent; OR, odds ratio; T2DM, type 2 diabetes mellitus.

## Discussion

Good glycemic control (HbA1c<7%) was achieved in 41.7% of patients enrolled in our study. In Jordan, another study conducted by Khattab *et al*.^17^ found that 35% of type 2 diabetic patients had HbA1c less than 7%. She also found that increased duration of diabetes, not following an eating plan, negative attitude toward diabetes and increased barriers to adherence scale score were all significantly associated with increased odds of poor glycemic control.

Many other studies have also found an alarming number of type 2 diabetic patients with uncontrolled DM and have reported a closer and similar percentage of uncontrolled HbA1c^18^–^20^. Despite the fact that only 41.7% of our studied population achieved HbA1c less than 7%, this percentage is higher than the ones reported by other studies. For example, Noureddine *et al*.^21^ reported that only 31.8% of type 2 diabetic patients attained HbA1c less than 7%. Xu *et al*.^22^ in a cross-sectional survey in North-Western China found that only 25.9% of type 2 DM patients included in this study achieved good glycemic control. Moreover, Yusufali *et al*.^23^ in Dubai, also reported that only 33% of type 2 DM patients who were screened had HbA1c less than 7%. On the other hand, Yokoyama *et al*.^24^ in a large scale survey in Japan reported that HbA1c less than 7% was achieved in 52.9% of type 2 DM patients.

In the present study, 15.4% of patients could achieve simultaneous control of HbA1c, BP and LDL-C. Schroeder *et al*.^25^, in a retrospective cohort study from 2000 to 2008, reported a higher rate of simultaneous control of 16–30%. At the same time, Xu *et al*.^22^ reported a much lower rate, with only 4.5% of patients attaining simultaneous control of the ABCs of diabetes.

In the present study, patients with poor glycemic control were more likely to be prescribed a combination of OHAs and insulin, indicating the need for higher doses or additional treatment as an attempt to provide better glycemic control for the deteriorations of diabetes over time. Consistent with this finding, Goudsward *et al*.^26^, Al-Nuaim *et al*.^27^, and Valle *et al*.^28^ also found the association between poor glycemic control with a combination of OHAs and insulin as a treatment for type 2 DM, reflecting the fact that more progressive disease will require more aggressive treatment combination to provide better glycemic control.

We found that poor glycemic control was more common among obese patients. In agreement with our findings, Hu *et al*.^19^ also reported that obesity is one of the factors associated with poor glycemic control. In addition, Quah *et al*.^29^ found that obesity was related to poor glycemic control.

This study showed that longer duration of diabetes (5–10 years or >10 years) was associated significantly with poor glycemic control and this is possibly explained by the progressive impairment in insulin secretion that will eventually end by pancreatic failure with increasing DM duration. This finding is consistent with that reported by other studies^17^,^20^,^30^–^32^.

In the present study, uncontrolled HbA1c level was significantly higher among diabetic patients with retinopathy. Consistent with our finding, Ahmed *et al*.^33^ also reported that retinopathy was significantly associated with poorly controlled diabetes. Many other studies have documented the close association between high HbA1c and diabetic retinopathy^34^–^40^.

The current study showed that the overall prevalence of hypertension was 75.4%. Females had a more controlled level of BP, less than 140/90, compared to males. Consistent with our results, Peng *et al*.^41^ also reported an overall prevalence of hypertension of 74.8% among the Chinese population in Shanghai. Mubarak *et al*.^42^ also found a prevalence rate of hypertension of 72.4% among 1000 patients with type 2 DM attending a national diabetes center in Jordan. Male sex has been associated with an increased prevalence of uncontrolled BP in our study and some other studies^43^,^44^. Hypertension among type 2 DM appeared to be age-related. This age-related trend of hypertension is consistent with that reported in the research literature^41^,^42^,^45^–^47^. Our study also showed that overweight and obese patients have a higher risk of uncontrolled BP than patients with normal BMI. In agreement with our findings, Mubarak *et al*.^42^, Dyer *et al*.^43^, Wilson *et al*.^44^, Lauer *et al*.^48^, Sonne-Holm *et al*.^49^ and Bertoni *et al*.^50^ also found a significant association between greater BMI and uncontrolled hypertension.

Our study showed that the use of insulin was significantly associated with uncontrolled BP. Consistent with our finding, Persson 51 also reported that after 2 months of insulin use, a surprisingly uniform increase in BP values was observed. Singh *et al*.^52^ also found that noninsulin-dependent DM patients have a tendency to retain sodium under the influence of insulin. Despite the fact that insulin has a vasodilatory effect, it is also known to stimulate the sympathetic neurovascular system and promote renal sodium reabsorption. Many studies have shown that an increase in plasma insulin markedly reduces sodium excretion and increases levels of serum sodium, contributing to the elevation in BP. Increased body weight with the use of insulin could also be a major contributor to the increase in BP.

The present study has some limitations. The anthropometric measures and laboratory measures were not measured by the researcher. The main limitation is that it was based on the abstraction of data from medical records. Thus, many important variables, such as medication adherence or patient behavior, such as diet or physical exercise, were not assessed. Another limitation is that the odds ratio might be exaggerated for events with high frequencies, such hypertension, poor glycemic control, and dyslipidemia.

## Conclusion

The overall prevalence of poor glycemic control was high and alarming. Factors associated with poor glycemic control were obesity, DM duration between 5 and 10 years or more than 10 years, retinopathy, and the use of a combination of OHA plus insulin or insulin alone. Furthermore, the simultaneous control of HbA1c (<7%), BP (<140/90), and LDL (<100 mg/dl), collectively known as the ABCs of diabetes, was achieved in only 15.4% of our patients. Future research should focus on capturing all variables that may impact glycemic, BP, and dyslipidemia control, with special emphasis on a healthy lifestyle that would be of great benefit in this control

## Ethical approval

The study was approved by the Ethical Committee at the National Center for Diabetes Endocrinology and Genetics (NCDEG), which is accredited by the National Ethics Committee. The study was conducted in accordance with the Declaration of Helsinki. This study was approved by the Institutional Review Board at NCDEG. Confidentiality has been assured to patients.

## Patient consent

An informed written consent was obtained from each participant. The confidentiality of the information was assured and only used for scientific purposes.

## Source of funding

The authors have no financial relationship relevant to this article to disclose.

## Author contribution

D.H.: wrote the manuscript, was the originator of the manuscript subject, and supervised the research; N.A.N.: helped in developing the idea, setting the protocol, supervised, and edited the manuscript; Q.A.: collected the data; Y.J: helped in developing the idea; A.M: contributed in writing the manuscript, acquisition of data, analysis, and interpretation of data; Y.K.: performed the statistical analysis, approved the protocol from a statistical point of view, analyzed the data, and approved the results; O.F.: helped in the statistical analysis; M.E-K.: contributed to the conception and design of the study and was responsible for lab analyses; K.A.: originator of the manuscript subject, supervised the research, and helped in developing the idea.

## Conflicts of interest disclosure

The authors declare that they have no conflicts of interest.

## Research registration unique identifying number (UIN)

1. Name of the registry: a study of the control of diabetes, hypertension, and dyslipidemia in a developing country (Jordan).
2. Unique identifying number or registration ID: researchregistry7406.
3. Hyperlink to your specific registration (must be publicly accessible and will be checked): not applicable.

## Guarantor

Prof Kamel Ajlouni.

## Provenance and peer review

Not commissioned, externally peer-reviewed.
